# Conserved Omp85 lid-lock structure and substrate recognition in FhaC

**DOI:** 10.1038/ncomms8452

**Published:** 2015-06-10

**Authors:** Timm Maier, Bernard Clantin, Fabian Gruss, Frédérique Dewitte, Anne-Sophie Delattre, Françoise Jacob-Dubuisson, Sebastian Hiller, Vincent Villeret

**Affiliations:** 1 Biozentrum, University of Basel, Klingelbergstr. 70, Basel 4056, Switzerland; 2 CNRS UMR8576, Unité Glycobiologie Structurale et Fonctionnelle, Villeneuve d'Ascq 59658, France; 3 Univ. Lille Nord de France, Lille, France; 4 Institut Pasteur de Lille, Center for Infection and Immunity, Lille 59021, France; 5 CNRS UMR8204, Lille 59046, France; 6 INSERM U1019, Lille 59045, France

## Abstract

Omp85 proteins mediate translocation of polypeptide substrates across and into cellular membranes. They share a common architecture comprising substrate-interacting POTRA domains, a C-terminal 16-stranded β-barrel pore and two signature motifs located on the inner barrel wall and at the tip of the extended L6 loop. The observation of two distinct conformations of the L6 loop in the available Omp85 structures previously suggested a functional role of conformational changes in L6 in the Omp85 mechanism. Here we present a 2.5 Å resolution structure of a variant of the Omp85 secretion protein FhaC, in which the two signature motifs interact tightly and form the conserved ‘lid lock'. Reanalysis of previous structural data shows that L6 adopts the same, conserved resting state position in all available Omp85 structures. The FhaC variant structure further reveals a competitive mechanism for the regulation of substrate binding mediated by the linker to the N-terminal plug helix H1.

## Abstract (web summary)

The fundamental processes of protein insertion and translocation at the outer membrane are mediated by Omp85 proteins. Here the authors report structures of the translocase FhaC, showing that the critical L6 loop adopts a conformation similar to that of related insertases; establishing a common structural basis for Omp85 function.

## Graphical abstract

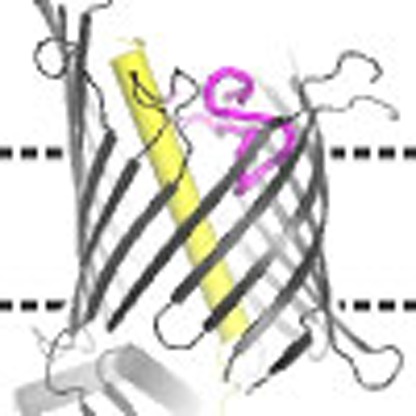

Omp85 proteins, such as the general *Escherichia coli* insertase BamA and the mitochondrial Sam50, are responsible for the insertion of β-barrel membrane proteins into the outer membranes of Gram-negative bacteria, mitochondria and chloroplasts^1^,^2^,^3^. In Gram-negative bacteria, Omp85 family members also act as translocases (TpsB) for the secretion of specific partner proteins (TpsA) across the outer membrane^4^,^5^. Such two-partner-secretion (Tps) systems are commonly contributing to bacterial pathogenicity^6^,^7^. One of the best-studied Tps systems comprises the translocase FhaC and its substrate filamentous hemagglutinin (FHA), which is functionally involved in virulence and biofilm formation in *Bordetella pertussis*^8^. Crystallographic structure determination of FhaC has provided the first depiction of the general Omp85 architecture^9^. This architecture builds upon a C-terminal, membrane-integrated β-barrel with 16 strands and up to six N-terminal POTRA domains directly attached to it. In FhaC, the two POTRA domains are involved in substrate recognition^10^, while the barrel forms a translocation pore for FHA secretion^11^. The FhaC pore in its crystallized state is plugged with an N-terminal helical extension, the H1 helix, which is absent in Omp85 insertases but generally found in TpsB transporters.

The mechanism of Omp85-mediated protein insertion had remained enigmatic until the recent structure determinations of bacterial BamA and TamA^12^,^13^,^14^. BamA and TamA are characterized by an unusually weak connection between their β-strands 1 and 16, which facilitates unzipping and inward kinking of strand 16. A lipid head group occupies the position of the displaced strand in the 2.3 Å crystal structure of TamA, demonstrating the formation of a gate towards the lipid phase^12^. Molecular dynamics simulations of BamA recognized a distorted lipid bilayer around the kink and suggest that the initial gate promotes further unzipping up to a complete lateral barrel opening^14^. The functional requirement for unzipping of strands 1 and 16 was elegantly demonstrated for BamA by disulfide bond trapping^15^.

The barrels of BamA and TamA are tightly closed on the extracellular side by a lid, which is mainly formed by the large extracellular loop L6. This arrangement brings the two most conserved sequence motifs in the entire Omp85 family in close contact. These motifs are located at the tip of L6, and in the central inner barrel wall, respectively^12^,^14^,^16^,^17^. To emphasize the nature of the strong interactions between these two motifs and its role for the lid conformation, the emerging structural feature has been termed the ‘lid lock'^12^. Despite the pronounced sequence conservation, the conformation of the L6 loops in the Omp85 insertases is drastically different from the one observed in the crystal structure of FhaC, where loop L6 reaches to the periplasmic face of the barrel^9^.

On the basis of the available structural insights, several models have been formulated for the Omp85 mechanism^18^, involving lateral gating^12^,^14^, hybrid barrel formation^12^,^19^, alteration of the lipid bilayer structure^14^,^20^ and conformational switching of POTRA domains and the L6 lid loop for pore opening and substrate transport^9^,^14^. Here we present the 2.5 Å resolution structure of the FhaC variant (V169T,I176N), which is characterized by disrupted substrate recognition and referred to as ‘FhaC_DIS_' (disruption). The data provide evidence for the general conservation of the L6 loop conformation, and thus a new perspective on the role of the lid-lock arrangement in pore opening and substrate translocation. The mutant structure resolves interactions of the H1 plug helix and its linker with the pore and the POTRA domains as a structural basis for the dynamic plugging mechanism of FhaC.

## Results and discussion

### Intermolecular helix swap in the FhaC_DIS_ crystal structure

**Figure 1:**
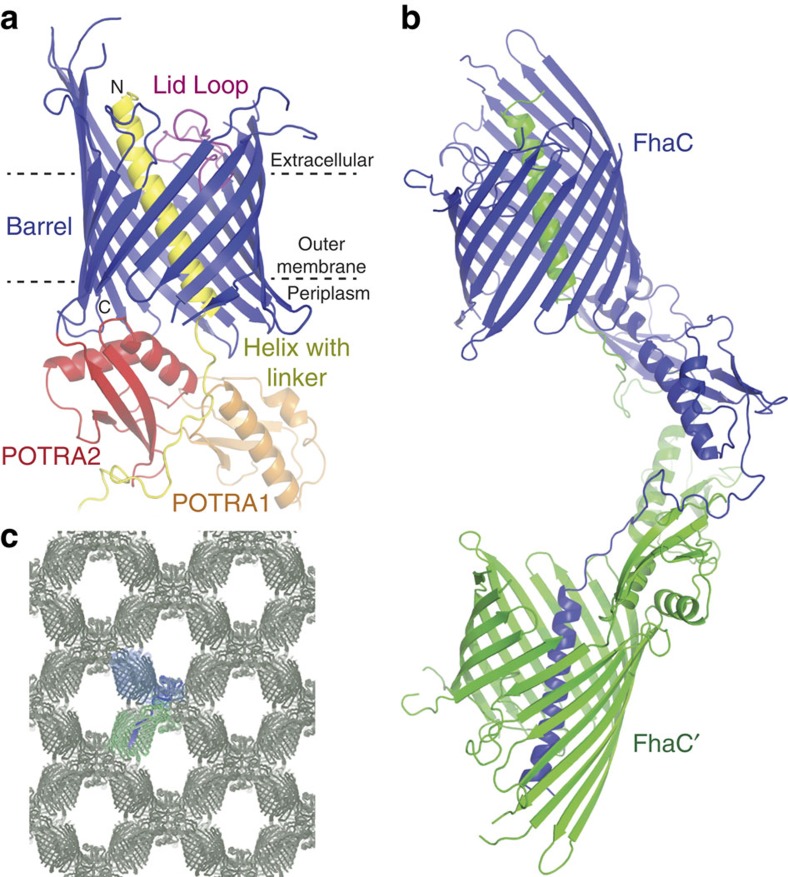
Structure and crystal packing of FhaC_DIS_. (**a**) Structural overview. Individual protein domains are colour-coded: Helix H1 with linker, yellow; POTRA1, orange; POTRA2, red; Barrel, blue; Lid Loop, magenta. (**b**) Formation of a swapped dimer in crystals of FhaC_DIS_ by exchange of helix H1. The two crystallographically related protein molecules involved in swapping are coloured blue and green, respectively. (**c**) Crystal packing of FhaC_DIS_. Two molecules equivalent to **b** are shown in the respective colours.

FhaC_DIS_ carries two point mutations, V169T and I176N, which locate to the POTRA2 domain and abolish the secretion activity of FhaC due to a disruption of POTRA2–substrate interaction^10^. FhaC_DIS_ was crystallized from micelles in space group C222_1_, isomorphous to wild-type (WT) FhaC crystals, but with considerably better diffraction than previous WT- or mutant FhaC crystals^9^,^16^. The structural model of FhaC_DIS_ was refined to R_work_/R_free_ of 21.7%/25.9% at 2.5 Å resolution (Table 1). It comprises the N-terminal extension helix H1, connected via a 25-residue linker to two POTRA domains and a C-terminal 16-stranded β-barrel (Fig. 1a). In contrast to previous FhaC structures^9^,^16^, the register of the H1 helix was unambiguously determined from side-chain electron density. The polypeptide linker between POTRA1 and helix H1 in FhaC_DIS_ was found to be well ordered. Tracing of the linker revealed that in the current crystal structure two adjacent FhaC_DIS_ molecules form an intermolecularly swapped dimer via exchange of their helices H1 (Fig. 1b,c).

The FhaC_DIS_ structure triggered a reanalysis of the previous 3.15 Å resolution WTFhaC data, which were reprocessed from raw images to 2.9 Å resolution using state-of-the-art techniques. Reinterpretation of the WTFhaC diffraction data on the basis of the FhaC_DIS_ structure clearly revealed modelling ambiguities in the previous WTFhaC structure (PDB entry 2QDZ^9^, referred to as ‘WTFhaC_old_') and yielded a revised model of WTFhaC (referred to as ‘WTFhaC_new_').

### Comparison of FhaC structural models: H1 linker and L6 loop

**Figure 2:**
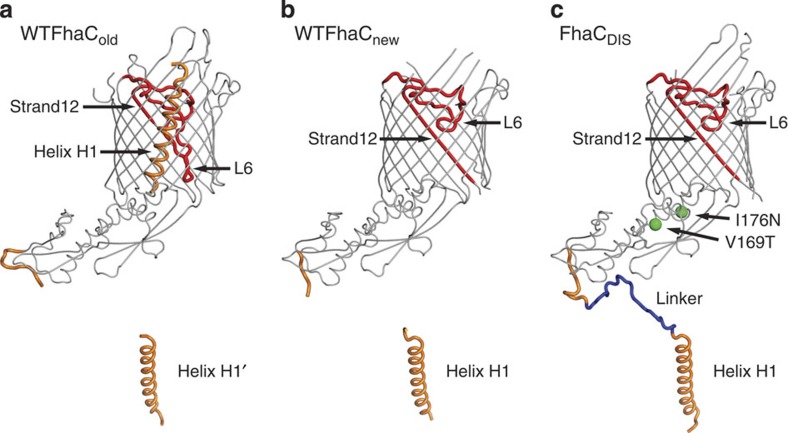
Overall comparison of FhaC_DIS_ and WTFhaC structural models. The structural models of (**a**) superseded WTFhaC_old_, (**b**) WTFhaC_new_ and (**c**) FhaC_DIS_ are shown in backbone representation. Strand12 and lid loop L6 (red), helix H1 and its linker (orange, blue for the linker segment disordered in WTFhaC_new_) are shown thick and in colour. For the superseded WTFhaC_old_ structure, the symmetry-related helix H1' is shown to indicate that it has the same position as H1 in WTFhaC_new_ and FhaC_DIS_. For FhaC_DIS_, the locations of the V169T and I176N mutations are indicated by green spheres.

**Figure 3:**
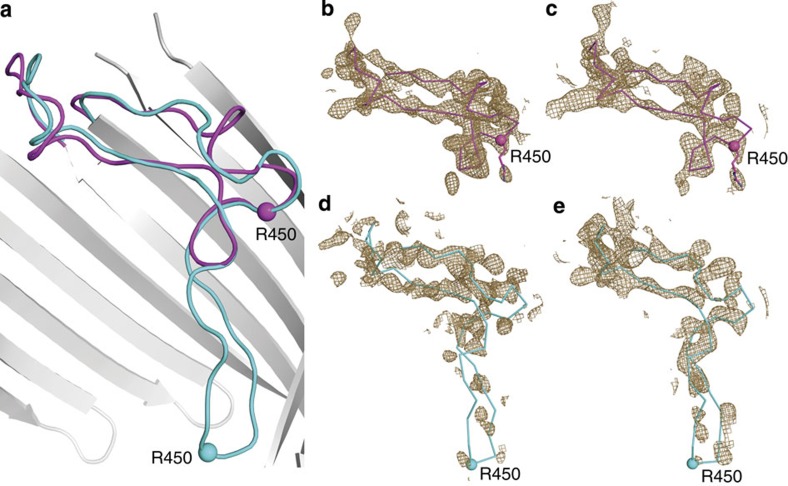
Comparison of L6 conformations in FhaC_DIS_ and WTFhaC_old_. (**a**) Superposition of L6 loop conformations in the superseded WTFhaC_old_ structure (cyan, PDB entry 2QDZ^9^) and the FhaC_DIS_ structure (magenta, PDB entry 4QL0; this work). Grey: The barrel of the FhaC_DIS_ structure. (**b**–**e**) Simulated annealing composite omit maps for different combinations of structures and diffraction data. Maps are shown at 1.5σ contour level in a radius of 2.5 Å around atoms belonging to the L6 loop and are calculated based on (**b**) FhaC_DIS_ model and the FhaC_DIS_ diffraction data. (**c**) FhaC_DIS_ model and WTFhaC diffraction data. (**d**) Superseded WTFhaC_old_ model and FhaC_DIS_ diffraction data. (**e**) Superseded WTFhaC_old_ model and WTFhaC diffraction data.

To clarify these differences, we compare the three structural models of WTFhaC_old_, WTFhaC_new_ and FhaC_DIS_ in Fig. 2a–c. The structural models feature relevant differences in three regions: (i) The register of helix H1 in FhaC_DIS_ and WTFhaC_new_ is shifted by three residues relative to the partly modelled H1 helix of WTFhaC_old_. (ii) The linker segment between POTRA1 and helix H1 (residues 33–58) is well-ordered in FhaC_DIS_, but largely disordered in WTFhaC_old_ and WTFhaC_new_. Due to the isomorphous nature of the crystals, the intermolecular swap of helix H1 between two crystallographically related molecules of FhaC is also the most likely explanation for the WTFhaC crystal packing and therefore the assignments of helix H1 and the symmetry-related helix H1' have been swapped in WTFhaC_new_ relative to WTFhaC_old_. (iii) The positioning and structure of loop L6 and the adjacent strand is well-resolved in FhaC_DIS_ in contrast to WTFhaC_old_. FhaC_DIS_ differs from WTFhaC_old_ by a massive register shift in loop L6 and the following strand 12 and a concomitant change in the position of the tip of loop L6 by 17 Å (Figs 2 and 3, Supplementary Fig. 1). In WTFhaC_old_, loop L6 was depicted in a conformation, where its tip touches the periplasmic rim of the barrel (cyan conformation in Fig. 3a), but in the 2.5 Å resolution FhaC_DIS_ structure the tip of loop L6 touches the inner side of the barrel wall at strand 13 (magenta conformation in Fig. 3a, Supplementary Fig. 2). In particular, this ‘lid lock' conformation of loop L6 describes the WTFhaC data unambiguously better than the periplasmic conformation in WTFhaC_old_, as directly evidenced by omit maps calculated for these two conformations against the respective data sets (Fig. 3b–e) and has thus been adopted in WTFhaC_new_. We discuss the relevance of the L6 loop positioning for FhaC and Omp85 biology in general in the following.

### A conserved lid-lock structure in the Omp85 family

**Figure 4:**
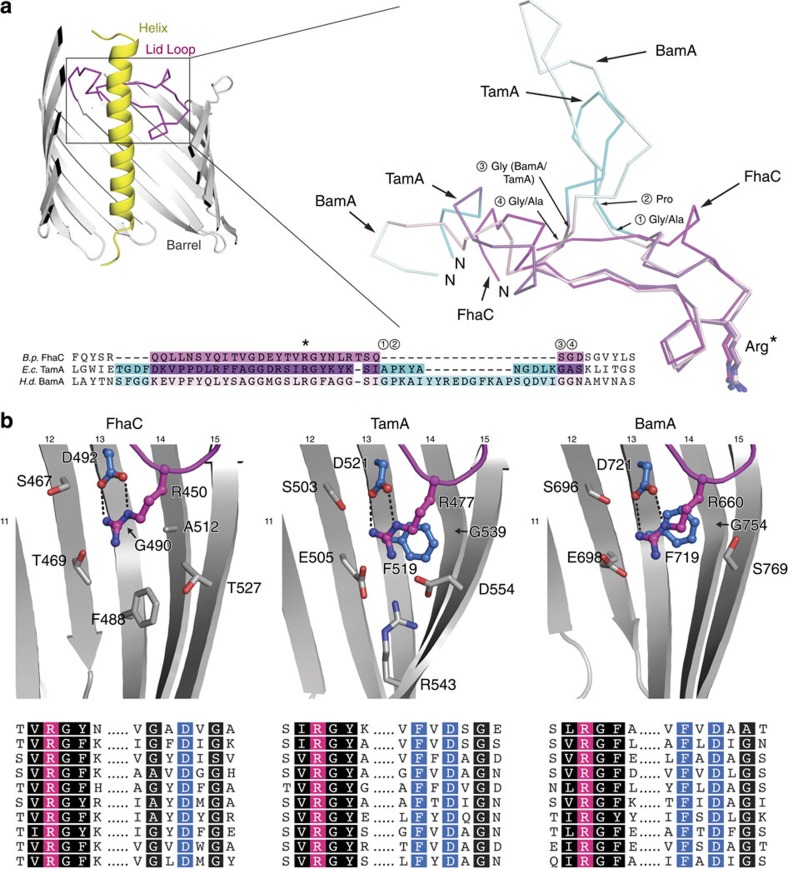
Conformation of loop L6 in the Omp85 family. (**a**) Cross-section through FhaC and structural and sequence alignment of L6 in *B. pertussis* FhaC (PDB entry 4QL0; this work), *E. coli* TamA (PDB entry 4C00^12^) and *H. ducreyi* BamA (PDB entry 4K3C^14^). Yellow: helix, grey: barrel. Conserved regions of L6 are shown in magenta, purple and light magenta for FhaC, TamA and BamA, respectively; loop extensions in TamA and BamA in cyan and light cyan, respectively. (**b**) Variations in the signature motifs correlate to barrel shape alterations in FhaC (left), TamA (center) and *H. ducreyi* BamA (right). Loop L6 and the barrel are coloured magenta and grey, respectively. Selected side chains are shown as sticks, highly conserved residues as ball-and-stick. Lower panel: Sequence alignments of signature motifs for 10 representative members of the FhaC (TpsB), TamA and BamA families, respectively (see Methods). The conserved arginine of the (V/I)**R**G(Y/F) motif is highlighted by magenta, the conserved interacting aspartate and phenylalanine of the (G/F)xDxG by blue. Other motif residues are indicated in black.

In the WTFhaC_new_ and the FhaC_DIS_ structures, the two signature motifs (V/I)**R**G(Y/F) at the tip of the loop L6 and (F/G)x**D**xG in the inner barrel wall on strand 13 are in close spatial contact and interact directly via a salt bridge between the conserved central residues **R**450 and **D**492. This ‘lid lock' formation is closely related to those previously found for TamA and BamA (Fig. 4a,b)^12^,^13^,^14^.

Interestingly, the arrangement of the entire lid loop is not even affected by the presence of a variable insertion site in loop L6, which can incorporate up to 30 additional amino-acid residues in TamA and BamA. In *B. pertussis* FhaC, the insertion region contains only two residues, which structurally bridge the insertion point in the shortest possible way (Fig. 4a). In *Haemophilus ducreyi* BamA and *E. coli* TamA, the insertion site is flanked by glycine or alanine residues, which permit a sharp outward kinking of the entire insert region (Fig. 4a, Supplementary Fig. 3). In consequence, the entire conserved regions of the L6 loop adopt almost identical conformations as demonstrated by very low backbone rmsd values (FhaC:TamA: 1.21 Å, FhaC:BamA: 1.37 Å; insertions excluded). The L6 loop is thus structurally conserved in all structures of Omp85 known so far, including the BamA insertase and the FhaC translocase, strongly suggesting a general functional role of the ‘lid lock' region for protein translocation and insertion in the entire Omp85 family.

### Structural variations around the lid lock

**Figure 5:**
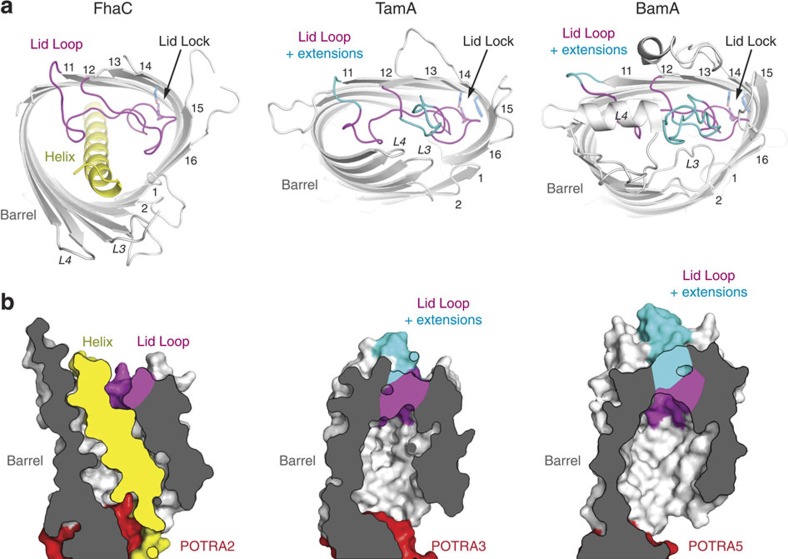
Barrel shape, lid lock and H1 helix insertion in FhaC. (**a**) Top-view ribbon representation of the barrels of *B. pertussis* FhaC (left; PDB entry 4QL0; this work), *E. coli* TamA (center; PDB entry 4C00^12^) and *H. ducreyi* BamA (right; PDB entry 4K3C^14^). The β-barrels are shown grey, the helix of FhaC is shown yellow. Using the same colour code as in Fig. 4, conserved regions of loop L6 are shown in magenta, extensions of L6 in TamA and BamA relative to FhaC are shown in cyan. (**b**) Cross-sectional surface representation of the barrels of *B. pertussis* FhaC (left), *E. coli* TamA (center) and *H. ducreyi* BamA (right), in the same colour code. POTRA domains are shown in red.

A key difference between FhaC and BamA/TamA is the presence of the N-terminal helix H1 in FhaC, which plugs the FhaC pore by traversing the entire length of the barrel. Two structural adaptations of FhaC relative to BamA/TamA pave the way for complete insertion of H1: first, the loops L3 and L4 are opened up in FhaC, whereas they tightly interact with L6 to close the extracellular face of the barrel in BamA/TamA (Fig. 5a,b). Second, FhaC features a wider and rounder barrel shape, which enlarges the pore at the periplasmic face relative to the kidney-shaped structures of BamA and TamA.

The FhaC_DIS_ and WTFhaC_new_ structures indicate an involvement of the lid-lock motif on strand 13 in barrel shape determination: The sequence motif on strand 13 has the form FxDxG in TamA/BamA, but GxDxG in FhaC proteins (Fig. 4b). In TamA and BamA insertases, the central arginine of the (V/I)**R**G(Y/F) motif stacks on top of the phenylalanine residue of the FxDxG motif. This phenylalanine side chain bends towards a conserved glycine residue of neighboring strand 14 (G539/G754 in TamA/BamA). Accommodation of the phenylalanine side chain requires a pronounced bend in strand 14, which correlates with a strongly curved region of the barrel.

In contrast, FhaC features a glycine as the first residue in its GxDxG motif and additionally, strand 14 contains a conserved alanine (A512) at the adjacent position (Fig. 4b). These two adaptations in FhaC permit lid-lock formation without requiring a bend of strand 14. They may contribute to an inherently expanded barrel shape, which prepares FhaC for helix H1 insertion, although a contribution of the H1 insertion itself to barrel shape alterations cannot be ruled out. The FhaC structure describes an Omp85 conformation, in which a polypeptide—not a native substrate, but the H1 plug helix—can traverse an Omp85 pore without release of the conserved lid-lock structure.

### Intermolecular swapping occurs in a defined linker region

Previous biochemical experiments have shown that the N-terminal helix H1 of FhaC acts as a dynamic plug, which spontaneously inserts into the barrel lumen of FhaC both in lipid vesicles and *in vivo* and occupies its central pore^21^. In the absence of substrates, helix H1 preferentially traverses the FhaC pore in a defined, rigid conformation and extends its N terminus into the extracellular space, while blocking channel activity. On substrate transport it is released from the pore and is flexibly posed on the periplasmic face of the membrane in the vicinity of the POTRA domains^21^. The interaction mode of a swapped dimer in the crystal structure of FhaC_DIS_ is not compatible with a native-like topology of the bacterial outer membrane (Fig. 1b,c). However, the unswapped form would correspond to a state of H1, which has all characteristics of the resting, plugged state of FhaC.

**Figure 6:**
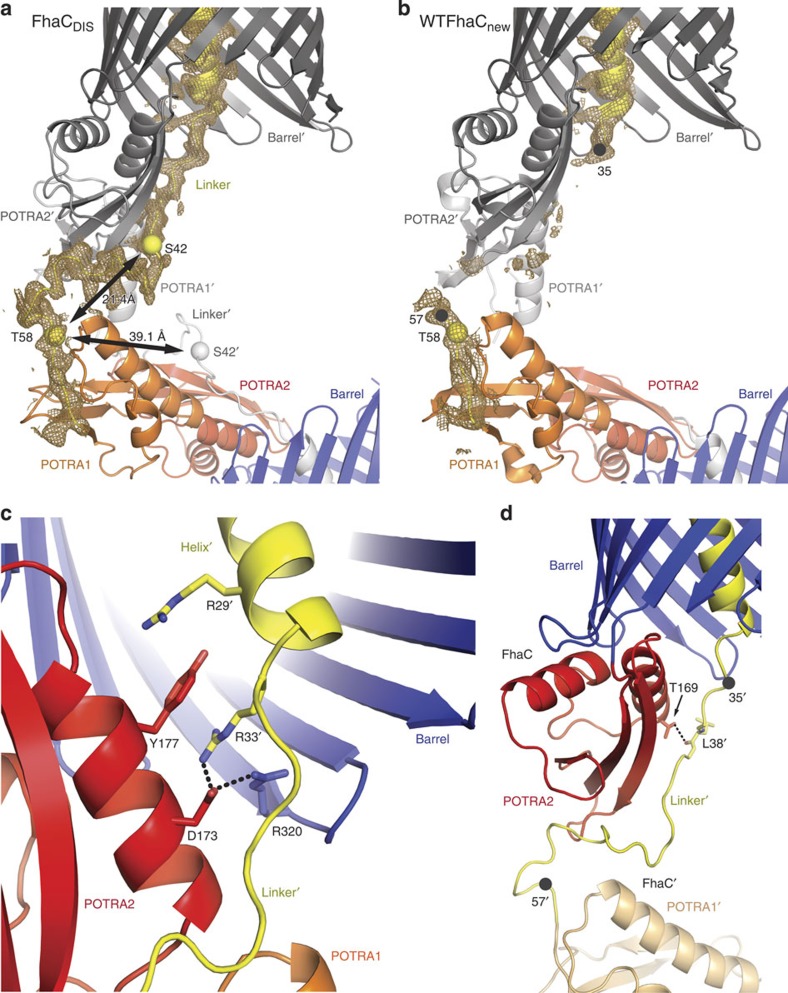
FhaC_DIS_ linker and helix H1 interactions. (**a**) 2FoFc map for FhaC_DIS_ shown in a radius of 2.5 Å around atoms belonging to the linker (yellow) at 1σ contour level. Residues Ser42 and Thr58, which encompass the domain-swapping segment are shown as yellow spheres. (**b**) 2FoFc map for WTFhaC shown in the same region as for FhaC_DIS_ at 1σ contour level. Residues 35 and 57, which encompass the disordered linker segment are shown as black spheres. (**c**) Interactions at the C-terminus of the helix including cation-π stacking and salt bridges. (**d**) Ribbon representation of the linker region in FhaC_DIS_ showing the hydrogen bond between Leu38 and the mutated Thr169. For comparison, the linker region disordered in WTFhaC is indicated by black spheres for residues 35 and 57.

From an inspection of the structure, we suggest that the swapping occurs in the 15 amino-acid region between residues 42 and 58 (Fig. 6), because all linker residues N terminal to this segment remain in direct interaction with POTRA2 and all linker residues C terminal to this segment remain in close contact to POTRA1 (Fig 6a,b). The role of residue 58 as a fixed anchor for the H1 linker agrees well with all available data: In reconstituted systems of FhaC transport, the mobility of residue 58 is not altered by substrate addition^21^ and in many FhaC-related TpsB proteins, residue 58 is mutated to a cysteine and presumably disulfide bonded to a nearby cysteine residue in POTRA1^21^ (Supplementary Fig. 3).

In the crystallographic intermolecularly swapped dimer, the distance between the two ends of the segment 42–58 is only 21 Å, which is spanned by the 15-residue polypeptide segment in a crouched conformation. The corresponding distance for an intramolecular H1-barrel interaction has a length of 39 Å, which could still be spanned by the 15-residue segment in a more extended conformation (Fig. 6a). Presumably, the shorter intermolecular swapping conformation was preferentially selected by crystal packing. The fact that the native membrane topology would readily prevent the existence of the swapped dimer configuration observed in the crystal, explains the lack of selective pressure on the linker segment to prevent such an arrangement.

### Competitive interplay of substrates and the H1 plug helix

The FhaC_DIS_ structure, together with published biochemical and biophysical studies, rationalizes the plugging mechanism of TpsB proteins. The H1 helix comprising residues 1–32 shows numerous interactions with the barrel wall, including 5 salt bridges, 12 hydrogen bonds and a total interaction interface of 988 Å^2^. Yet, this interaction interface almost completely lacks elements of side-chain interlocking, which would hinder a sliding of H1 along the barrel wall (Supplementary Fig. 4). The FhaC_DIS_ structure now points to a key role of the N-terminal residues 29–38 for defining H1 interactions (Fig. 6c). In this segment, Arg33 is positioned by a salt bridge to Asp173, providing an interdigitation of side chains by stacking between Tyr177 of POTRA2 and Arg320 at the periplasmic rim of the barrel. Arg29 of the terminal winding of H1 may contribute to this interaction by stacking to the other face of Tyr177.

In WTFhaC_new_, the polypeptide region 36–56 is disordered and thus remains structurally unresolved (Fig. 6a,b). In contrast, in FhaC_DIS_ carrying the (V169T,I176N) double mutation, the full linker is ordered. In fact, Leu38, the third residue of the segment that is disordered in WTFhaC_new_, directly interacts in FhaC_DIS_ with the mutated residue Thr169 by formation of a hydrogen bond between the Leu38 backbone-N and the Thr169 side chain-OH (Fig. 6d). Apparently, this interaction triggers ordering also of the entire segment, where residues 39 to 42 are involved in further interactions with the same POTRA2 domain.

**Figure 7:**
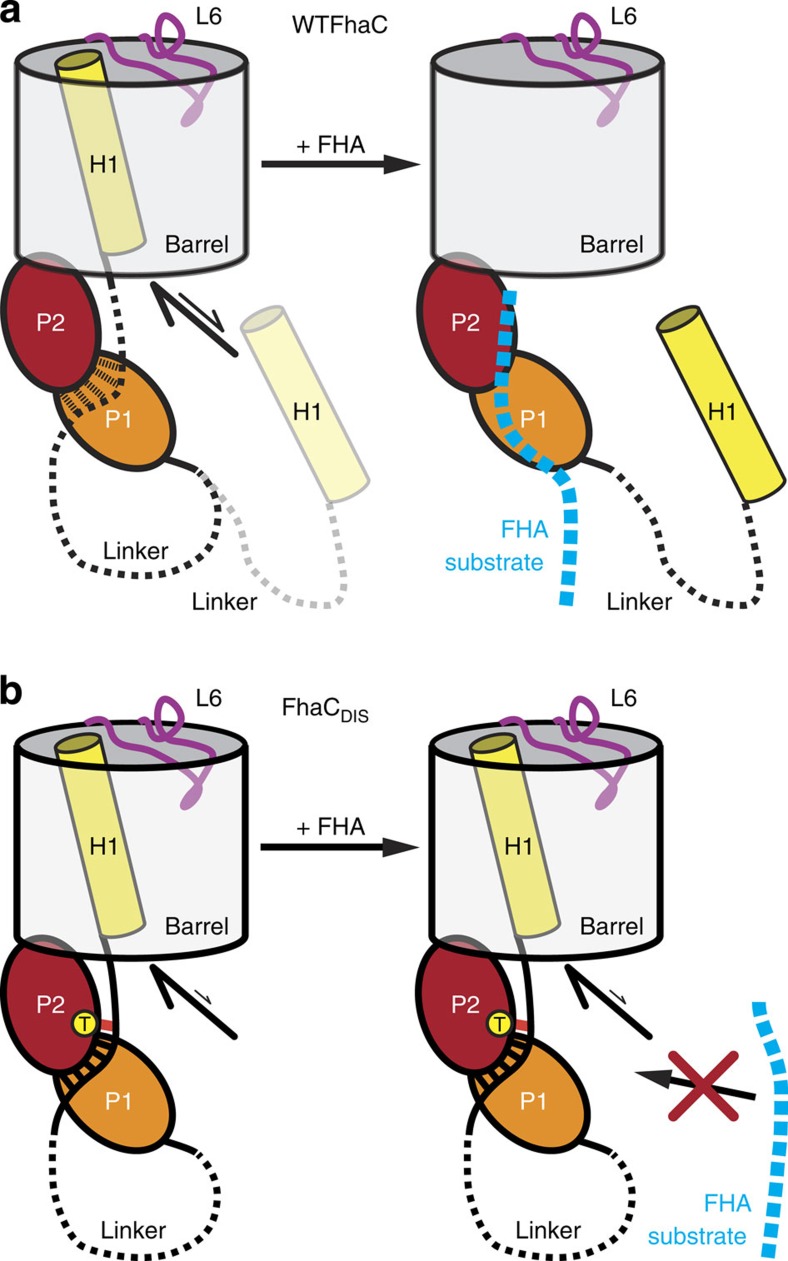
Plug helix H1 release mechanism of FhaC. (**a**) In WTFhaC, the linker interacts weakly with POTRA domain 2, and the helix is preferentially inside the barrel. On substrate arrival, FHA competes with the linker for interaction with POTRA2, extruding the helix from the barrel and allowing the substrate to be transported. (**b**) In FhaC_DIS_, the introduced hydrogen bond between Leu38 and Thr169 leads to stronger interactions between the linker and POTRA2, with which an arriving substrate cannot compete and thus will not be transported.

Biochemical data clearly indicate that the observed interactions around residues 33 and 38 (Fig. 6c,d) play a key role for the functional mechanism of FhaC. The double mutation (V169T,I176N), which was designed to interfere with β-augmentation substrate interactions of POTRA2, not only abolishes transport, but directly interferes with substrate interaction^10^; it thus affects a direct substrate binding site. We hypothesize that also in WTFhaC, the linker region 33–38 interacts at least transiently with the region around residues V169 and I176 on POTRA2. The H1 linker could thus be displaced by substrate binding, resulting in a destabilization of the contacts that lock the position of helix H1 to the barrel around residue Arg33. Indeed, EPR-based mobility assays on membrane-inserted FhaC have demonstrated a direct effect of substrate interaction on the mobility of residue 33 (ref. 21): This residue is mostly immobile in the absence of a substrate and even remains partly immobile on deletion of the entire downstream H1 helix, demonstrating an inherent H1-independent component of tethering to POTRA1. In both cases, with and without H1, substrate addition drastically increases the mobility of residue 33. Altogether, this mechanism directly couples substrate binding to a preferential release of helix H1 into the periplasm (Fig. 7).

While recent structural and functional studies have provided important insights into Omp85 function, the underlying principles of the distinct translocase and insertase function remain unknown. The crystal structure of FhaC_DIS_ now demonstrates a general structural conservation of the signature motifs in the lid-lock region in translocases and insertases of the Omp85 family. It also correlates variations in the signature sequences to the wider barrel shape of FhaC, which permits passage of the plug helix H1 all the way through the pore while the lid lock is formed.

Altogether with detailed previous studies on substrate interaction and FhaC mobility, the FhaC_DIS_ structure reveals a competitive mechanism for coupling of substrate recognition and plug helix release. Most TpsB proteins are predicted to harbour a helical segment followed by a disordered region at the N terminus^21^. It is therefore probable that the mechanism revealed by this study will prove generally relevant to two-partner secretion. FhaC and related Tps systems play a prominent role in bacterial pathogenesis; identification of the competitive plugging mechanism may ultimately expose a novel target site for fighting bacterial infection.

## Methods

### Protein production and purification

The plasmid pFJD138–V169T–I176N, which encodes FhaC_DIS_ with an N-terminal 6-His tag^10^, was used to produce FhaC_DIS_ for crystallization experiments. *E. coli* BL21(DE3)-omp5 transformed with pFJD138–V169T–I176N were grown at 37 °C in liquid LB broth to an absorbance of 1 (A_600_) and protein expression was induced overnight at 20 °C with 1 mM IPTG. Cells were collected, washed in 20 mM sodium phosphate (pH 7) and resuspended in the same buffer containing 0.01 mg ml^−1^ DNase and a mixture of protease inhibitors (Roche). Cells were broken by passages through a French pressure cell. After collecting the membrane fractions by ultracentrifugation (100,000 × *g* for 1 h), two steps of extraction were performed successively with 0.8 and 1.5% β-octyl glucoside. The second extract was subjected to chromatography onto a cation-exchange column Poros HS20 (Perkin-Elmer) equilibrated in 20 mM sodium phosphate (pH 7.0) with 1% β-octyl glucoside. FhaC was eluted with a linear 0–1 M gradient of NaCl. The FhaC-containing fractions were pooled and applied onto a 1 ml HiTrap chelating column (Amersham Biosciences) equilibrated in 20 mM sodium phosphate (pH 7.0), 1% β-octyl glucoside. FhaC was eluted by a pulse of 500 mM imidazole (pH 6.5) in the equilibration buffer. For crystallization, FhaC was concentrated to 26 mg ml^−1^ by using Vivaspin centrifugal devices with a 50 kDa cut-off (Vivascience).

### Crystallization and data collection

Crystals were obtained at 20 °C using the hanging drop vapour diffusion method. The protein and precipitant solutions were mixed in a 1:1 ratio. Crystals were grown at a protein concentration of 26 mg ml^−1^ in 34% PEG1000, 1% β-octyl glucoside and 500 mM imidazole (pH 6.5). Diffraction data were collected at 100 K on beamline ID14-4 at the European Synchrotron Radiation Facility (Grenoble, France). All diffraction data were processed with XDS^22^. Data collection and refinement statistics are summarized in Table 1. Whereas the crystal packing remained isomorphous to the old crystal form, the diffraction limit increased to 2.5 Å. Model building was accomplished manually with Coot (Crystallographic Object Oriented Toolkit)^23^ from the CCP4 suite^24^. The refinement with Buster^25^ led to an *R*_work_ of 21.7% and an *R*_free_ of 25.9% using data to 2.5 Å for FhaC_DIS_ and *R*_work_/*R*_free_ of 22.2%/27.9% using data to 2.9 Å for the reprocessed WTFhaC data^9^. The final model for the FhaC_DIS_ structure lacks the first two N-terminal residues, residues 296 and 297 of extracellular loop L3, 381 to 399 of L5, 478 to 481 of periplasmic turn T6, 499 to 503 of L7 and residues 537 and 538 of L8. Loop L6 and the linker between the helix and POTRA1 are well defined in the electron density. Furthermore, a total of three detergent molecules were well ordered and bound to FhaC. In addition, three PEG molecules were found inside the barrel and two PEG molecules are located close to the periplasmic pore in vicinity of the POTRA domain anchor. The updated WTFhaC_new_ model lacks the first five N-terminal residues, residues 36–56 of the linker connecting the helix and POTRA1, residues 294–301 of L3, 342–350 of L4, 381–399 of L5, 475–481 of T6, 500–502 of L7 and 533–543 of L8. All structural differences between the FhaC_DIS_ model and the superseded WTFhaC_old_ model (PDB entry 2QDZ^9^) are summarized in Supplementary Table 1. Composite omit maps after simulated annealing of the FhaC_DIS_ structure and the superseded WTFhaC_old_ structure against the FhaC_DIS_ and the WTFhaC diffraction data (PDB entry 2QDZ^9^) were generated using PHENIX^26^.

### Sequence alignments

Eleven TamA orthologs, ten BamA orthologs and ten TpsB proteins were selected from the NCBI database, showing pairwise sequence identities between 18 and 36% within each group^12^ (accession codes P0ADE4.1, WP_010374432.1, YP_006917734.1, YP_005378779.1, WP_006914415.1, WP_006956461.1, WP_007639592.1, YP_006416500.1, YP_007468392.1, WP_008316497.1, YP_006721763.1, YP_002998039.1, YP_001121414.1, WP_003783125.1, YP_001219350.1, WP_010501263.1, YP_865762.1, YP_007459313.1, YP_004865655.1, YP_002549812.1, WP_008996841.1, AAB30624.1, YP_335961.1, WP_005764711.1, YP_003741556.1, WP_002831157.1, YP_006646915.1, YP_004122309.1, WP_008291755.1, WP_005980414.1, YP_003307097.1). In addition, the *H. ducreyi* BamA sequence was added (NCBI accession code 4K3C_A) and a TpsB sequence (WP_004649222.1). Alignments, starting with the second last POTRA domains, were performed with Clustal Omega^27^,^28^ and further edited taking into consideration available structural data (Supplementary Fig. 3).

## 

## Additional information

**Accession codes**: Atomic coordinates and structure factors have been deposited in the Protein Data Bank with accession code 4QL0 for FhaC_DIS_, and 4QKY for WTFhaC_new_.

**How to cite this article:** Maier, T. *et al*. Conserved Omp85 lid-lock structure and substrate recognition in FhaC. *Nat. Commun.* 6:7452 doi: 10.1038/ncomms8452 (2015).

## Supplementary Material

Supplementary Information Supplementary Figures 1-4, Supplementary Table 1 and Supplementary References

## Figures and Tables

**Table 1:** Data collection and refinement statistics.

	WTFhaC_new_	FhaC_DIS_
*Data collection*		
* *Space group	C 2 2 21	C 2 2 21
Cell dimensions		
* a*, *b*, *c* (Å)	108.60, 136.65, 112.27	106.38, 136.95, 110.97
* *α, β, γ (°)	90, 90, 90	90, 90, 90
Resolution (Å)	50–2.90 (2.95–2.90)*	50–2.50 (2.55–2.50)
* R*_sym_ or *R*_merge_	0.090 (0.847)	0.057 (0.886)
* I*/σ*I*	19.88 (3.28)	23.04 (2.13)
* *CC(1/2) (%)	99.8 (75.6)	99.9 (68.9)
* *Completeness (%)	98.8 (98.3)	99.7 (99.9)
* *Redundancy	10.90 (10.70)	4.91 (4.93)
		
*Refinement*		
* *Resolution (Å)	39.34–2.90	41.95–2.50
* *No. reflections	18,626	28,332
* R*_work_/*R*_free_	0.222/0.279	0.217/0.259
No. atoms	3,731	4,474
Protein	3,726	4,095
Ligand/ion	5	304
Water	—	75
		
*B-factors*		
Protein	91.4	78.9
Ligand/ion	78.8	77.6
Water	—	63.8
		
*R.m.s. deviations*		
Bond lengths (Å)	0.010	0.010
Bond angles (°)	1.09	1.08

*Values in parentheses are for highest-resolution shell.
