# Supplementary material for: Conserved Omp85 lid-lock structure and substrate recognition in FhaC
Source: Nat Commun. 2015 Jun 10;6:7452. doi: 10.1038/ncomms8452 (PMC4490367; doi:10.1038/ncomms8452)
Supplement: Supplementary Information — Supplementary Figures 1-4, Supplementary Table 1 and Supplementary References [file ncomms8452-s1.pdf]

Supplementary Material for

Conserved Omp85 lid lock structure and substrate recognition in FhaC

Timm Maier[1#], Bernard Clantin[2,3#], Fabian Gruss[1], Frédérique Dewitte[2,3], Anne-Sophie Delattre[3,4,5,6], Françoise Jacob-Dubuisson[3,4,5,6], Sebastian Hiller[1], Vincent Villeret[2,3]

[#] These authors contributed equally

[1] Biozentrum, University of Basel, Klingelbergstr. 70, 4056 Basel, Switzerland

[2] CNRS UMR8576, Unité Glycobiologie Structurale et Fonctionnelle, Villeneuve d'Ascq 59658, France

[3] Univ. Lille Nord de France, Lille 59044, France

[4] Institut Pasteur de Lille, Center for Infection and Immunity, Lille 59019, France

[5] CNRS UMR8204, Lille 59021, France

[6] INSERM U1019, Lille 59045, France

Correspondance should be addressed to:

Timm Maier (timm.maier@unibas.ch), Sebastian Hiller (Sebastian.hiller@unibas.ch), Vincent Villeret (vincent.villeret@univ-lille1.fr)

This file includes:

Supplementary Figures 1-4
Supplementary Table 1
Supplementary References 1-3

Supplementary Figures

a

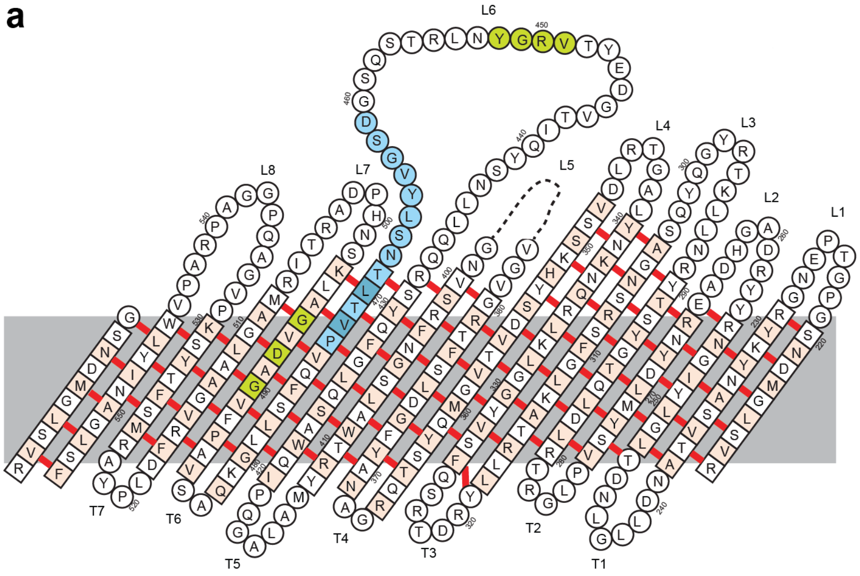

b

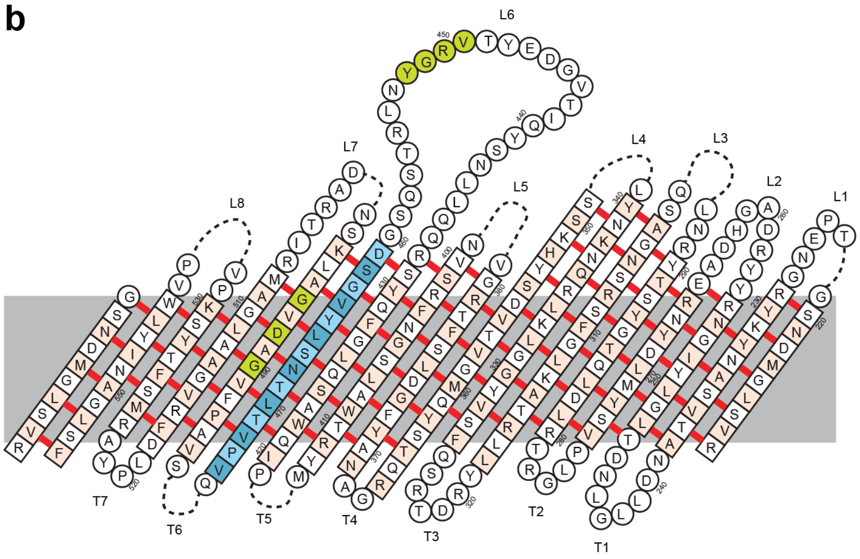

Supplementary Figure 1. Comparison of β-strand topologies in models of FhaC. 2D topology representations of (a) the superseded wtFhaC$_{old}$ structure (PDB entry 2QDZ[1]) and (b) the structures of FhaC$_{DIS}$ (PDB entry 4QL0; this work) and wtFhaC$_{new}$ (PDB entry 4QKY; this work) as viewed from the barrel exterior. Residues forming β-strands are indicated by rectangles, residues of loops and turns by circles. The side chains of orange residues point towards the membrane. The extracellular loops are numbered L1 to L8 from N- to C-terminus, the periplasmic turns are numbered T1 to T7. Interstrand backbone hydrogen bond pairs are indicated by red lines between the corresponding residues. Strand 1 is repeated on the left hand side to show the hydrogen bonds that form to strand 16. The conserved motifs (I/V)RG(Y/F) (residues 449–452) and GxDxG (residues 490–494) are highlighted green. Residues of strand 12 are highlighted blue to show the register shift that appeared in the superseded wtFhaC$_{old}$ structure[1] due to wrong tracing of the L6 loop.

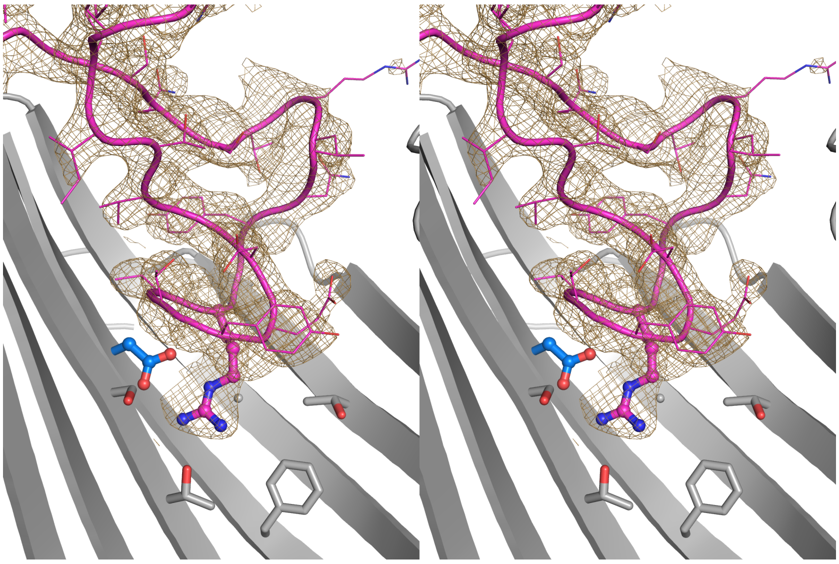

Supplementary Figure 2. Stereo view of electron density around the L6 loop of FhaC$_{DIS}$. The 2FoFc map for FhaC$_{DIS}$ is shown in a radius of 2 Å around atoms belonging to the L6 loop (magenta) at 1σ contour level. Side chains of the L6 loop are shown as lines, selected side chains of the barrel (grey) are shown as sticks, and highly conserved residues as ball-and-stick.

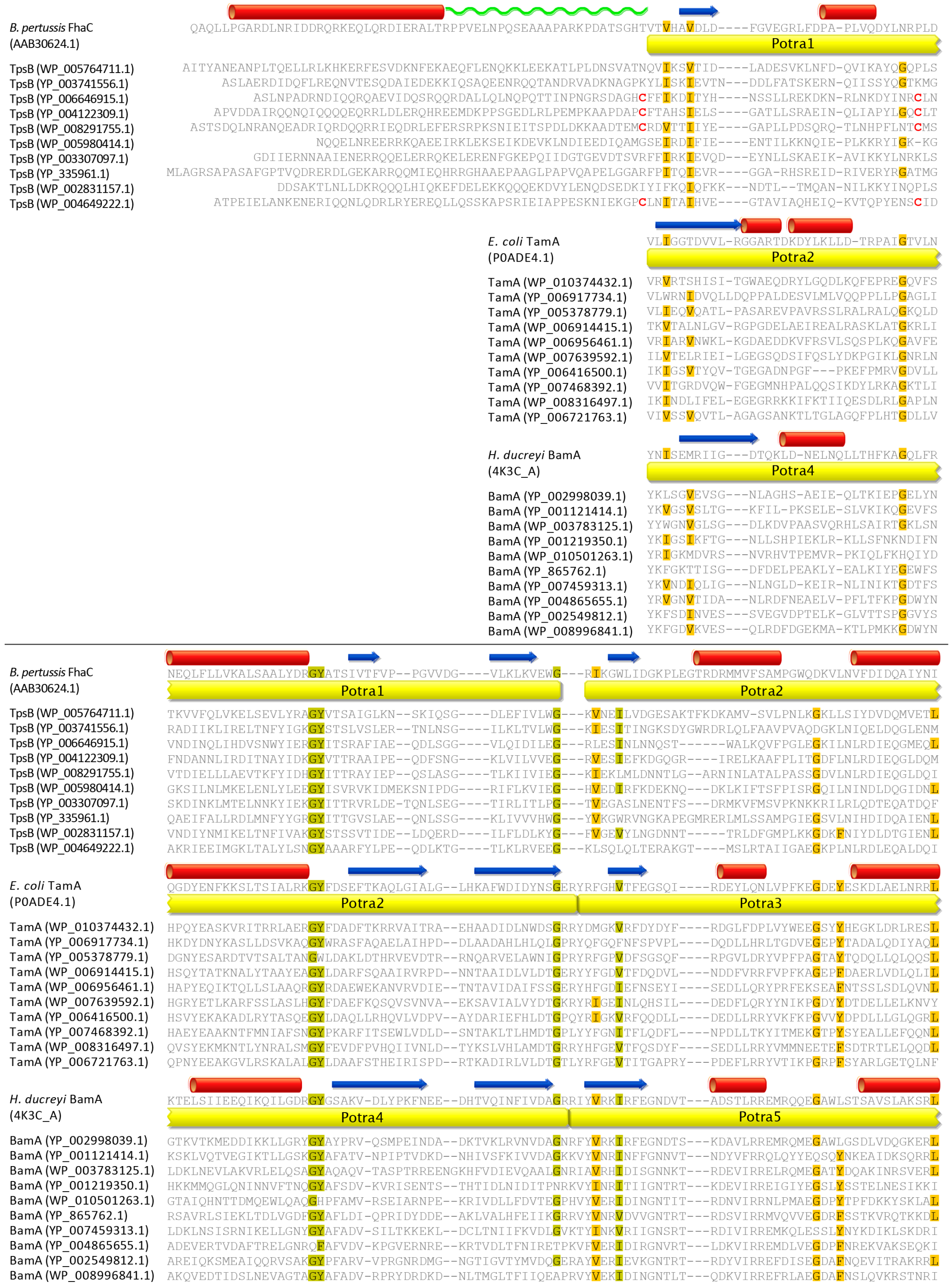
B. pertussis FhaC (AAB30624.1)
Potra1
TpsB (WP_005764711.1)
TpsB (YP_003741556.1)
TpsB (YP_006646915.1)
TpsB (YP_004122309.1)
TpsB (WP_008291755.1)
TpsB (WP_005980414.1)
TpsB (YP_003307097.1)
TpsB (YP_335961.1)
TpsB (WP_002831157.1)
TpsB (WP_004649222.1)
E. coli TamA (P0ADE4.1)
Potra2
TamA (WP_010374432.1)
TamA (YP_006917734.1)
TamA (YP_005378779.1)
TamA (WP_006914415.1)
TamA (WP_006956461.1)
TamA (WP_007639592.1)
TamA (YP_006416500.1)
TamA (YP_007468392.1)
TamA (WP_008316497.1)
TamA (YP_006721763.1)
H. ducreyi BamA (4K3C_A)
Potra4
BamA (YP_002998039.1)
BamA (YP_001121414.1)
BamA (WP_003783125.1)
BamA (YP_001219350.1)
BamA (WP_010501263.1)
BamA (YP_865762.1)
BamA (YP_007459313.1)
BamA (YP_004865655.1)
BamA (YP_002549812.1)
BamA (WP_008996841.1)
Potra1
Potra2
Potra2
Potra3
Potra4
Potra5

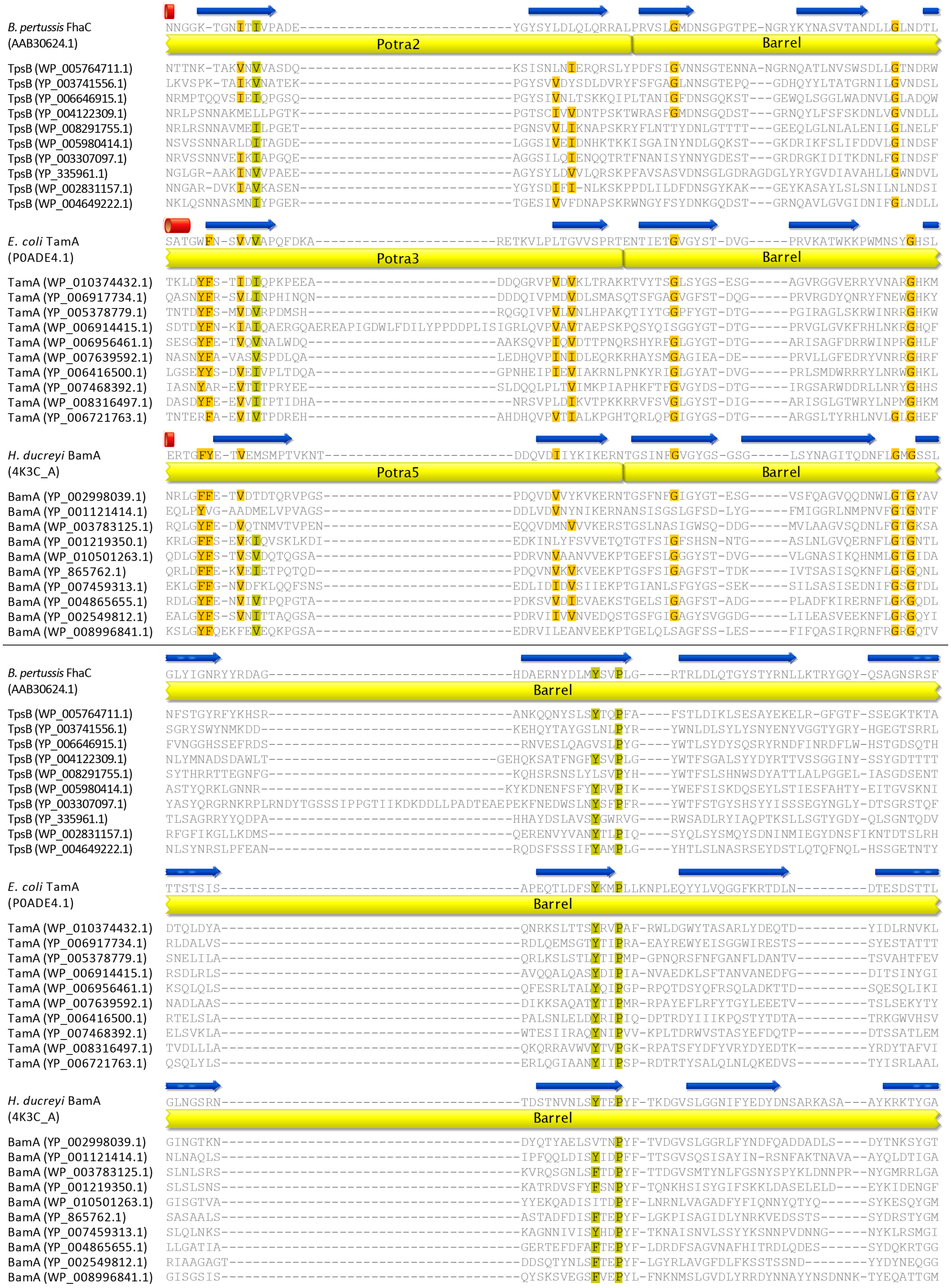

B. pertussis FhaC
(AAB30624.1)
Potra2
Barrel
TpsB (WP_005764711.1)
TpsB (YP_003741556.1)
TpsB (YP_006646915.1)
TpsB (YP_004122309.1)
TpsB (WP_008291755.1)
TpsB (WP_005980414.1)
TpsB (YP_003307097.1)
TpsB (YP_335961.1)
TpsB (WP_002831157.1)
TpsB (WP_004649222.1)
E. coli TamA
(P0ADE4.1)
Potra3
Barrel
TamA (WP_010374432.1)
TamA (YP_006917734.1)
TamA (YP_005378779.1)
TamA (WP_006914415.1)
TamA (WP_006956461.1)
TamA (WP_007639592.1)
TamA (YP_006416500.1)
TamA (YP_007468392.1)
TamA (WP_008316497.1)
TamA (YP_006721763.1)
H. ducreyi BamA
(4K3C_A)
Potra5
Barrel
BamA (YP_002998039.1)
BamA (YP_001121414.1)
BamA (WP_003783125.1)
BamA (YP_001219350.1)
BamA (WP_010501263.1)
BamA (YP_865762.1)
BamA (YP_007459313.1)
BamA (YP_004865655.1)
BamA (YP_002549812.1)
BamA (WP_008996841.1)

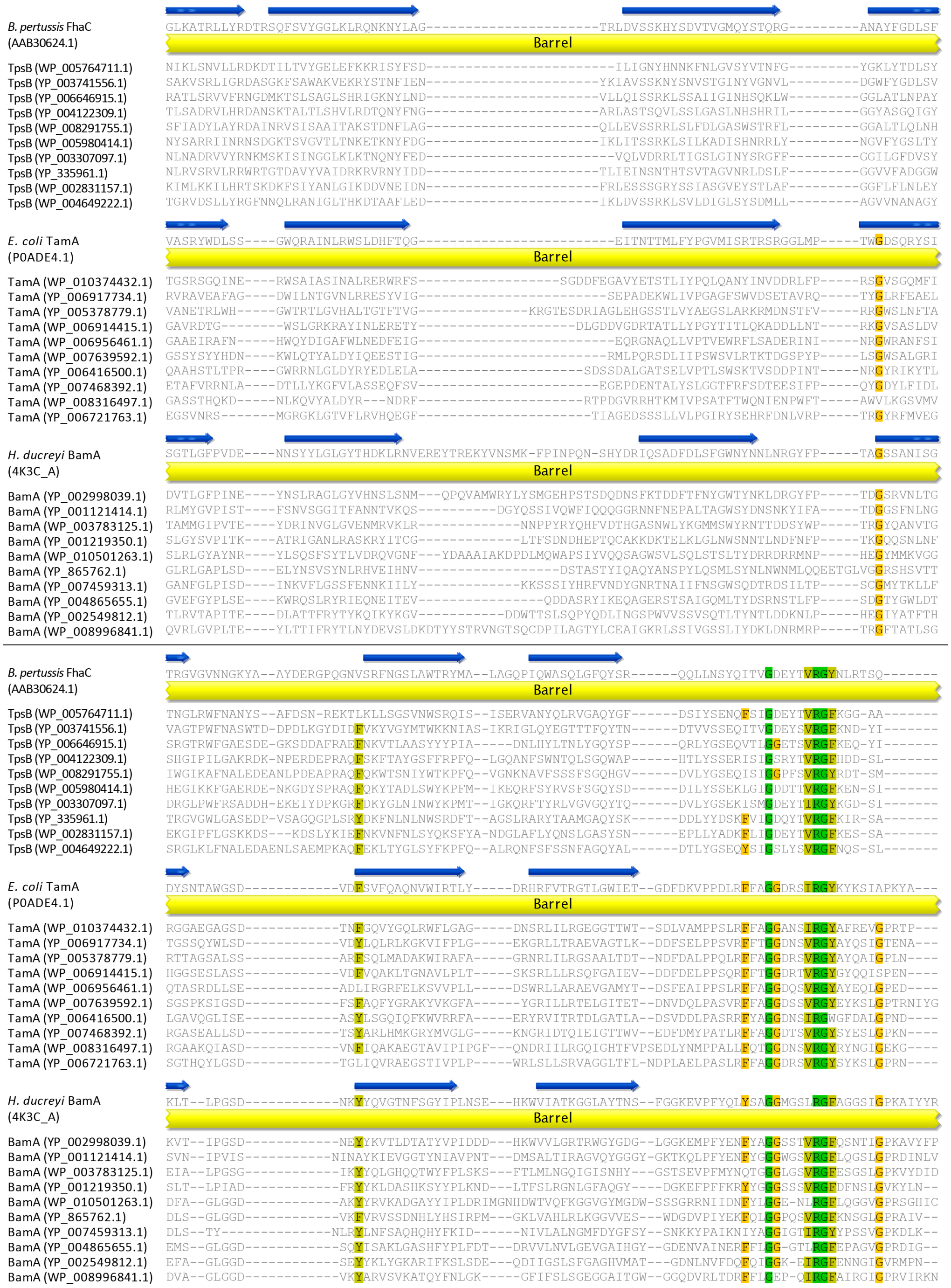
B. pertussis FhaC (AAB30624.1)
Barrel
TpsB (WP_005764711.1)
TpsB (YP_003741556.1)
TpsB (YP_006646915.1)
TpsB (YP_004122309.1)
TpsB (WP_008291755.1)
TpsB (WP_005980414.1)
TpsB (YP_003307097.1)
TpsB (YP_335961.1)
TpsB (WP_002831157.1)
TpsB (WP_004649222.1)
E. coli TamA (P0ADE4.1)
Barrel
TamA (WP_010374432.1)
TamA (YP_006917734.1)
TamA (YP_005378779.1)
TamA (WP_006914415.1)
TamA (WP_006956461.1)
TamA (WP_007639592.1)
TamA (YP_006416500.1)
TamA (YP_007468392.1)
TamA (WP_008316497.1)
TamA (YP_006721763.1)
H. ducreyi BamA (4K3C_A)
Barrel
BamA (YP_002998039.1)
BamA (YP_001121414.1)
BamA (WP_003783125.1)
BamA (YP_001219350.1)
BamA (WP_010501263.1)
BamA (YP_865762.1)
BamA (YP_007459313.1)
BamA (YP_004865655.1)
BamA (YP_002549812.1)
BamA (WP_008996841.1)

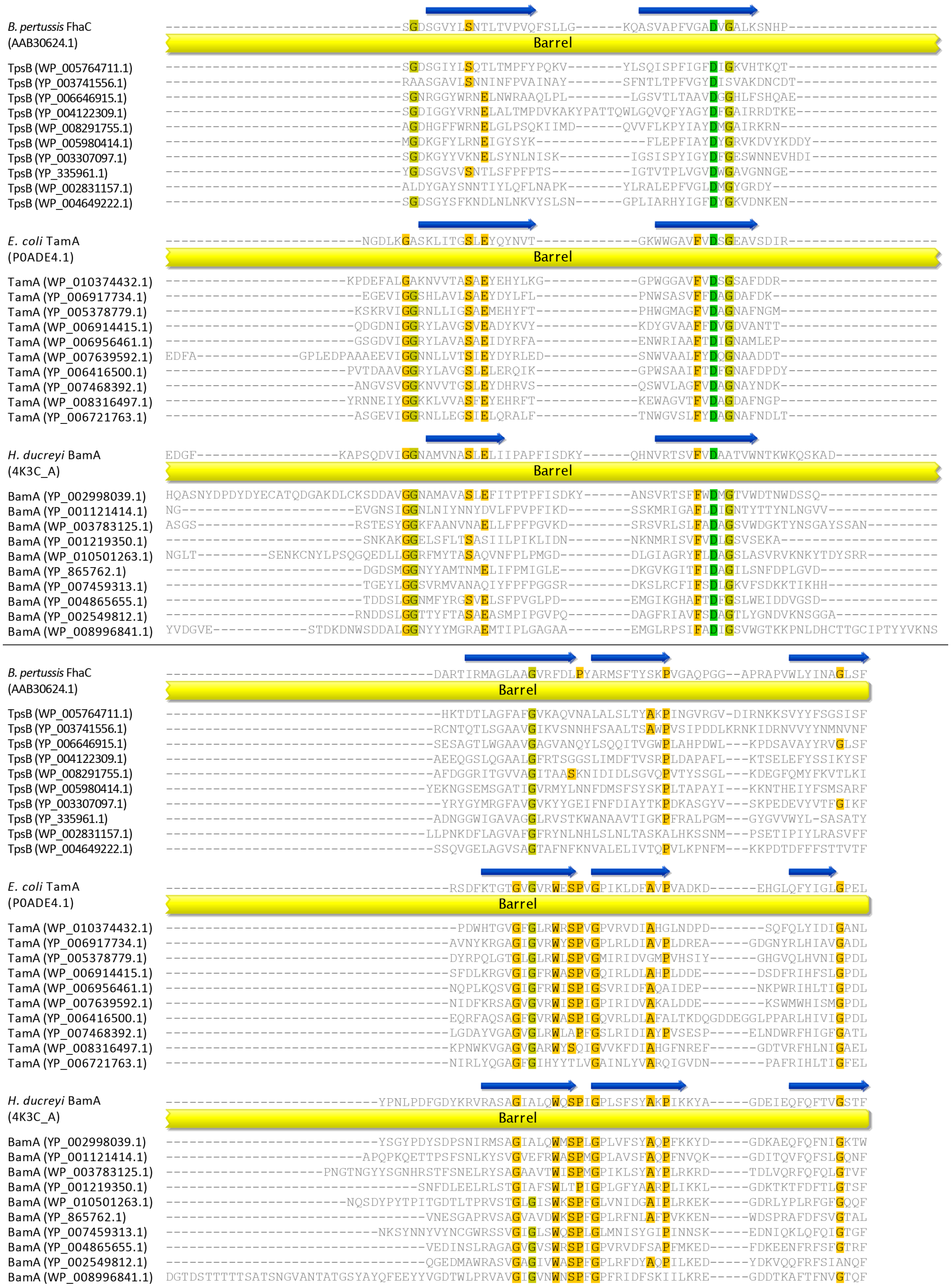

Supplementary Figure 3. Alignment of Omp85 sequences. 11 TpsB, 11 TamA and 11 BamA sequences beginning with the second last POTRA domains. For the known structures of *B. pertussis* FhaC (PDB entry 4QKY; this work), *E. coli* TamA (PDB entry 4C00[2]) and *H. ducreyi* BamA (PDB entry 4K3C[3]), α-helices and β-strands are indicated by red tubes and blue arrows, respectively, on top of the sequences. The sequences for the N-terminal helices and linkers of TpsB proteins are shown unaligned. Cysteines in the linker region and POTRA1 domain of TpsB proteins, plausibly forming disulfide bonds (see text), are highlighted in red.

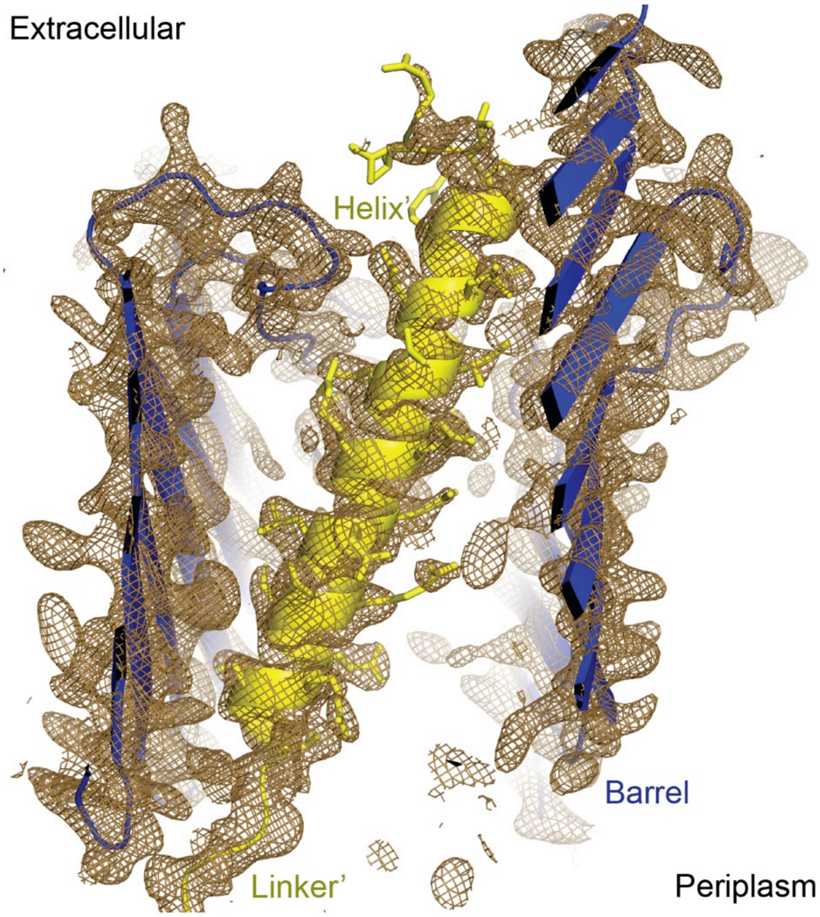

Supplementary Figure 4. Electron density around helix and barrel of FhaC$_{DIS}$. The 2FoFc map for FhaC$_{DIS}$ is shown in a radius of 2.5 Å around atoms belonging to helix and linker (yellow) and the barrel (blue) at 1σ contour level.

Supplementary Tables

Supplementary Table 1 Differences between FhaC structural models

Region	$FhaC_{DIS}$, residues	$wtFhaC_{old}$, corresponding residues	Variation
N-terminal helix	Gln3-Asp10	Gln3-Gly7	Residues from symmetry-related molecules in different conformations
N-terminal helix	Leu11-Arg33	Ala8-Ala30	Residues from symmetry-related molecules aligned with offset 3
Linker	Pro34-His57	Leu31-Asp52	Not built in superseded $wtFhaC_{old}$ model, well resolved in $FhaC_{DIS}$ structure
POTRA1	Thr58-Thr60	Ala53-Ser55	Aligned with offset 5
POTRA1	Val61	Gly56-Val61	Extra loop in $wtFhaC_{old}$ model
POTRA1	Val70-Glu71	Val70-Gly72	Different conformations
POTRA1	Gly72-Pro77	Arg73-Ala78	Aligned with offset -1
POTRA1	Ala78-Asp83	Pro79-Asp83	Different conformations
Barrel L1	Pro222-G228	Pro222-G228	Different conformations
Barrel L3	Leu294-Lys295	Leu294-Lys295	Different conformations
Barrel L3	Thr296-Arg297	The296-Arg297	Not built in $FhaC_{DIS}$ model
Barrel L4	Gly343-Leu346	Gly343-Leu346	Different conformations
Barrel L5	Val381-Val383	Val381-Val383	Not built in $FhaC_{DIS}$ model
Barrel L5	Gly398-Asn399	Gly398-Asn399	Not built in $FhaC_{DIS}$ model
Barrel L6	Arg432		Position skipped in $wtFhaC_{old}$ model
Barrel L6	Gln433-Asn437	Arg432-Leu436	Aligned with offset 1
Barrel L6	Ser438		Position skipped in $wtFhaC_{old}$ model
Barrel L6	Tyr439-Glu446	Asn437-Gly444	Aligned with offset 2
Barrel L6	Tyr447	Asp445-Thr456	Extra loop in $wtFhaC_{old}$ model
Barrel L6	Thr448-Gly451	Ser457-Gly460	Aligned with offset -9
Barrel L6	Tyr452-Thr456	Asp461-Val464	Different conformations
Barrel L6, S12	Ser457-Gln475	Tyr465-Ala483	Aligned with offset -8
Barrel T6	Phe476-Ser477		Positions skipped in $wtFhaC_{old}$ model
Barrel T6	Leu478-Lys481		Not built in $FhaC_{DIS}$ model, positions skipped in $wtFhaC_{old}$ model
Barrel T6	Gln482-Ala483		Positions skipped in $wtFhaC_{old}$ model
Barrel L7	Asn499-Ala503	Asn499-Ala503	Not built in $FhaC_{DIS}$ model
Barrel T7	Leu519-Pro520	Leu519-Pro520	Different conformations
Barrel L8	Gly533-Pro536	Gly533-Pro536	Different conformations
Barrel L8	Gly537-Gly538	Gly537-Gly538	Not built in $FhaC_{DIS}$ model
Barrel L8	Ala539-Ala542	Ala539-Ala542	Different conformations

Comparison of differences between the superseded $wtFhaC_{old}$ structure (PDB entry 2QDZ[1]) and $FhaC_{DIS}$ (PDB entry 4QL0; this work). Strands are indicated by "S", extracellular loops by "L" , periplasmic turns by "T".

Supplementary References

1. Clantin B*, et al.* Structure of the membrane protein FhaC: A member of the Omp85-TpsB transporter superfamily. *Science* **317**, 957-961 (2007).

2. Gruss F, Zähringer F, Jakob RP, Burmann BM, Hiller S, Maier T. The structural basis of autotransporter translocation by TamA. *Nat Struct Mol Biol* **20**, 1318-1320 (2013).

3. Noinaj N*, et al.* Structural insight into the biogenesis of β-barrel membrane proteins. *Nature* **501**, 385-390 (2013).
